# HME-FDM 3D-Printed Implantable Drug Delivery Systems-From Design to Characterization

**DOI:** 10.3390/pharmaceutics18080932

**Published:** 2026-07-29

**Authors:** Bence Vámosi, Ildikó Bácskay, Pálma Fehér, Zoltán Ujhelyi, Petra Arany

**Affiliations:** 1 Pharmacology and Microbiology Section, DE Doctoral School of Medical Sciences, H-4032 Debrecen, Hungary; vamosi.bence@pharm.unideb.hu; 2 Faculty of Pharmacy, Department of Pharmaceutical Technology, University of Debrecen, 4002 Debrecen, Hungary; bacskay.ildiko@pharm.unideb.hu (I.B.); feher.palma@pharm.unideb.hu (P.F.); 3 Faculty of Pharmacy, Department of Pharmaceutical Industry and Pharmaceutical Technology, University of Debrecen, 4002 Debrecen, Hungary; ujhelyi.zoltan@pharm.unideb.hu

**Keywords:** 3D printing, fused deposition modeling (FDM), hot-melt extrusion (HME), implantable drug delivery devices (IDDS), analytical testing, personalized medicine

## Abstract

Implantable devices have undergone enormous development in the past several decades and more results are expected as there are still unanswered questions. In manufacturing, a relatively new technology called three-dimensional (3D) printing has become increasingly involved, from which hot-melt extrusion (HME) coupled with fused deposition modeling (FDM) is one of the most researched methods in producing implantable drug delivery systems (IDDS). The HME process is used to produce polymer filaments that are the carriers of the applied drugs, while FDM creates the implant itself from the filaments, based on the computer-aided designs. The availability of several polymers like polycaprolactone, polylactic acid, or thermoplastic polyurethane, etc., allows the incorporation of many active pharmaceutical ingredients while digital designs offer numerous variabilities in designs, formulations, and applications of 3D-printed IDDS. This allows more specific and detailed modifications in the end product which can forecast the possibility of personalized treatments and therapies. In this review, our research group gathered together different HME-FDM-printed IDDS to provide examples about the diverse applicability of 3D printing. These products, just like any other device and medicine in the medical field, must be characterized and evaluated properly. The methods and technology that are needed already exist and can be repeatedly used in the characterization of IDDSs; we also discuss these methods based on the available publications. In conclusion, every condition is given to make research and manufacture personalized 3D-printed IDDSs possible, however more research work and proof of safe usage are crucial to make these devices applicable in everyday medical treatments.

## 1. Introduction

Implantable devices have gone through enormous development throughout the decades since the 1930s, when R. Deanesly and A.S. Parkes made the first implantable drug delivery system [1,2]. The primary objectives of implantable drug delivery devices are to maintain the concentration of the drug within the therapeutic interval for a longer period of time, avoiding multiple administration, improving patient adherence, while usually avoiding first-pass metabolism and enhancing comfort of the host [3,4,5]. As the basis for an implantable carrier system, several composition variations in polymers and hydrogels were developed to achieve the desired effect for well-defined treatments with many active pharmaceutical ingredients (APIs) [6]. The appearance of commercially available implants made sustained, predictable and continuous drug delivery possible, not just systemically, but even to targeted tissues, such as tumors [7]. Another benefit of using polymers is that some can degrade within the body to non-toxic monomers; this way, they can be eliminated by metabolism and there is no need for another invasive procedure to remove the implant [3]. Many forms and materials are compatible with the human body when it comes to implantable drug delivery devices or drug delivery systems, even containing electronic parts such as the implantable microchip-based human parathyroid hormone drug delivery device; however, non-metallic materials are dominating in this field when it comes to drug delivery systems [5].

Among the main disadvantages, the removal of the implant after use should be mentioned [8]. Sometimes it is considered more traumatic to remove the implant than the insertion itself. In addition, non-biodegradable polymers can accumulate in the tissue. Therefore, the evaluation of risks and benefits must be made during polymer selection [9]. The possible risk of implant contamination and disrupted drug delivery due to tissue damage are also not neglectable factors when balancing the advantages and disadvantages of implants. Therefore, individual cases might demand time-consuming consultations between other groups of professionals [10].

There are various methods of manufacturing polymer implants mentioning several techniques like compression, HME, or, as for porous polymer implants, particulate leaching, gas foaming, electrospinning, thermally induced phase separation, cryogelation, or freeze-drying [11,12]. The most commonly used, compression, has the advantage that it does not require heat nor solvent for manufacturing, but unluckily, the API dissolution can be quiet fast and irregular in shape and might cause inappropriate dissolution [9]. HME itself is a popular technique as it is a solvent-free, continuous, and scalable technique, but the API stability problems must be kept in mind [13]. Other newer techniques also face some advantages and some limitations [12]. Despite several manufacturing options, almost all of them are missing an important factor, personalization. A still rapidly developing method, additive manufacturing or 3D printing, could be a solution for this issue [14]. Three-dimensional printing technologies have many variations that use different kinds of materials, techniques, and geometries [15]. In the past several years, the most common additive manufacturing method in the pharmaceutical 3D printing research field is HME- FDM [16,17,18].

The characterization of these IDDSs is just as important as the manufacturing. Different characterization techniques are available for this purpose, such as physico-chemical characterization including powder X-ray diffraction (PXRD) or Fourier transform infrared spectroscopy (FTIR). Thermal characterization methods include thermogravimetric analysis (TG/TGA) and differential scanning calorimetry (DSC), while microscopic characterization methods like scanning electron microscopy (SEM), micro-computed tomography (micro-CT or μCT), X-ray computed tomography (XRCT), hot-stage microscopy (HSM), or polarized light microscopy (PLM) also provide valuable information about chemical stability, and behavior of materials during exposure to high temperatures. Some also enable the visualization of the integrity of the product or intermediate products [19].

In vivo and in vitro tests are also compulsory to determine the biocompatibility, potential cytotoxicity, biodegradability host response behavior, drug-dissolution profiles, and release rates of such devices [20].

In this study, the main principles of HME-FDM are gathered together on commonly used polymers. In a separate chapter, the HME-FDM manufacturing of implants is described in detail. The most important information is listed regarding the subtypes of the implants. In a separate chapter, the evaluation and the characterization are summarized. Then, some translational, regulations, limitations, and future prospectives are mentioned for better understanding.

## 2. Materials and Methods

This study was conducted based on a structured literature search to ensure transparency and reproducibility. Studies were identified through searches in the PubMed database and Google Scholar. Google Scholar was additionally used to retrieve peer-reviewed articles indexed in major scientific publishers, including Elsevier (ScienceDirect), Springer Nature, and MDPI. The search strategy employed combinations of keywords such as “HME”, “FDM”, “implantable drug delivery system”, “polymers”, “personalized medicine”, “analytical testing”, “controlled drug-release”, “3D printing”, “biocompatibility, “biodegradability”, “quality by design”, and “sterilization”. Although the search was mainly limited to articles published between 2016 and 2026, some works before 2016 were included. After removal of duplicates, studies were screened based on titles and abstracts for relevance. Full-text articles were then assessed for eligibility according to predefined inclusion and exclusion criteria. The inclusion criteria comprised HME-FDM-related processes, materials, software, and intermediate- and end-product characterization methods focusing on 3D-printed implantable drug delivery devices. Exclusion criteria included non-English publications, conference abstracts, reviews without original data, and studies lacking relevant biological outcomes. Although the study selection process followed PRISMA-inspired principles, this review remains narrative in design. Therefore, a formal systematic quality assessment and a PRISMA flow diagram were not performed. For illustration purposes, PTC Inc. Onshape (Version 1.207). PTC Inc.: Boston, MA, USA, 2025. Available online: onshape.com (accessed on 26 November 2025) and CraftWare (version 1.21.1, CraftUnique, Budapest, Hungary) were used.

## 3. Hot-Melt Extrusion (HME)

**Figure 1:**
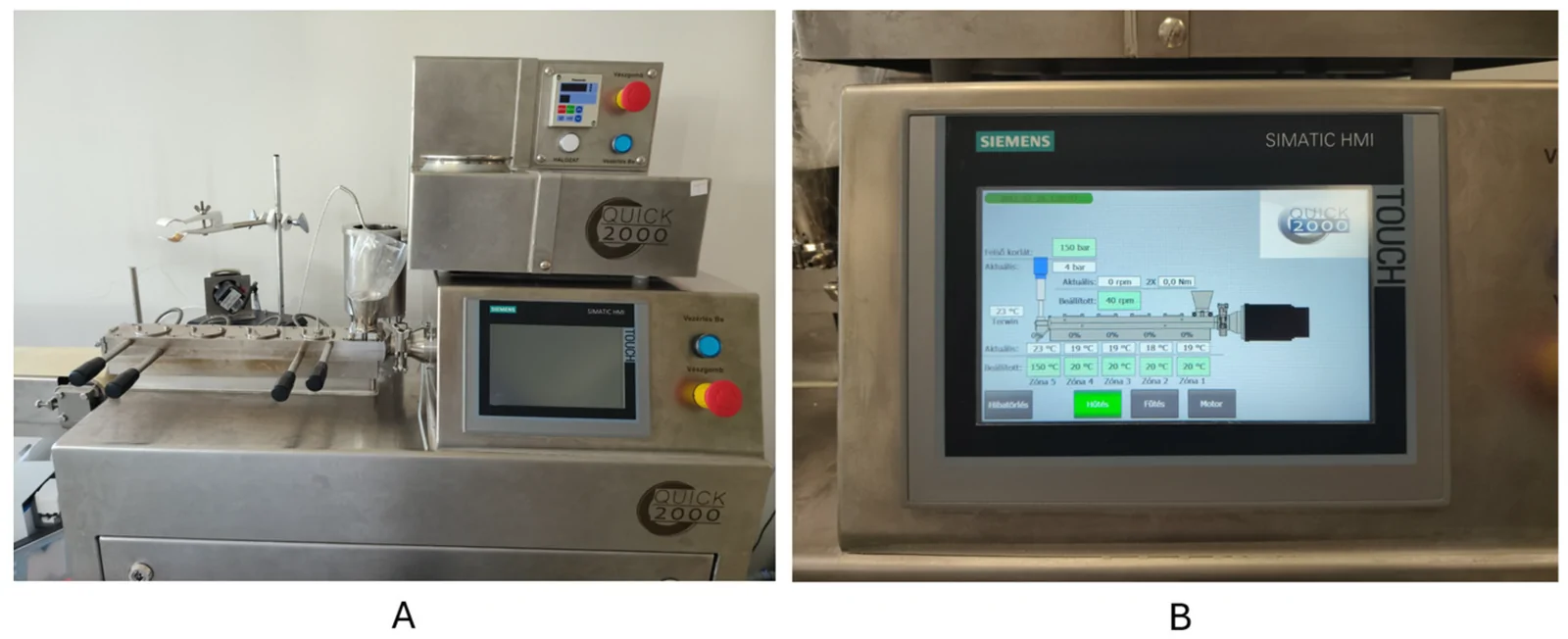
Twin-screw hot-melt extruder (**A**) and its control panel (**B**).

Hot-melt extrusion is a diverse technique which can also be used for API containing filament manufacturing coupled with FDM 3D printing. The preferred method of filament manufacturing is using a co-rotated twin-screw hot-melt extruder (Figure 1) [21,22]. The design of the twin-screw within the screw chamber makes it possible to mix and convey the added polymer and other excipients with the API into a homogeneous mixture while the applied heat and shear stress helps to melt and soften substances inside the twin-screw chamber [23]. The operating temperature, which can be set on the control panel of HME (Figure 1), must be in accordance with the selected polymer’s softening temperature range, otherwise the mixture might be clogged or burned before leaving the extruder’s nozzle [24,25]. After the homogeneous mixture leaves the nozzle, usually a conveyor belt drives the filament away from the nozzle and solidifies. The filament thickness is determined by the nozzle diameter and the conveying speed, which is synchronized with the screw speed, depending on the rheological properties of the semi-solid mixture. After solidification, it can be wound onto a spool for easier use and storage [22,26]. The viscoelastic properties of a polymer also influence the diameter of the filament due to the “die swell” behavior which means that the filament diameter will increase as it leaves the extruder [27].

## 4. Model Designing and Printing Process with FDM Printer

**Figure 2:**
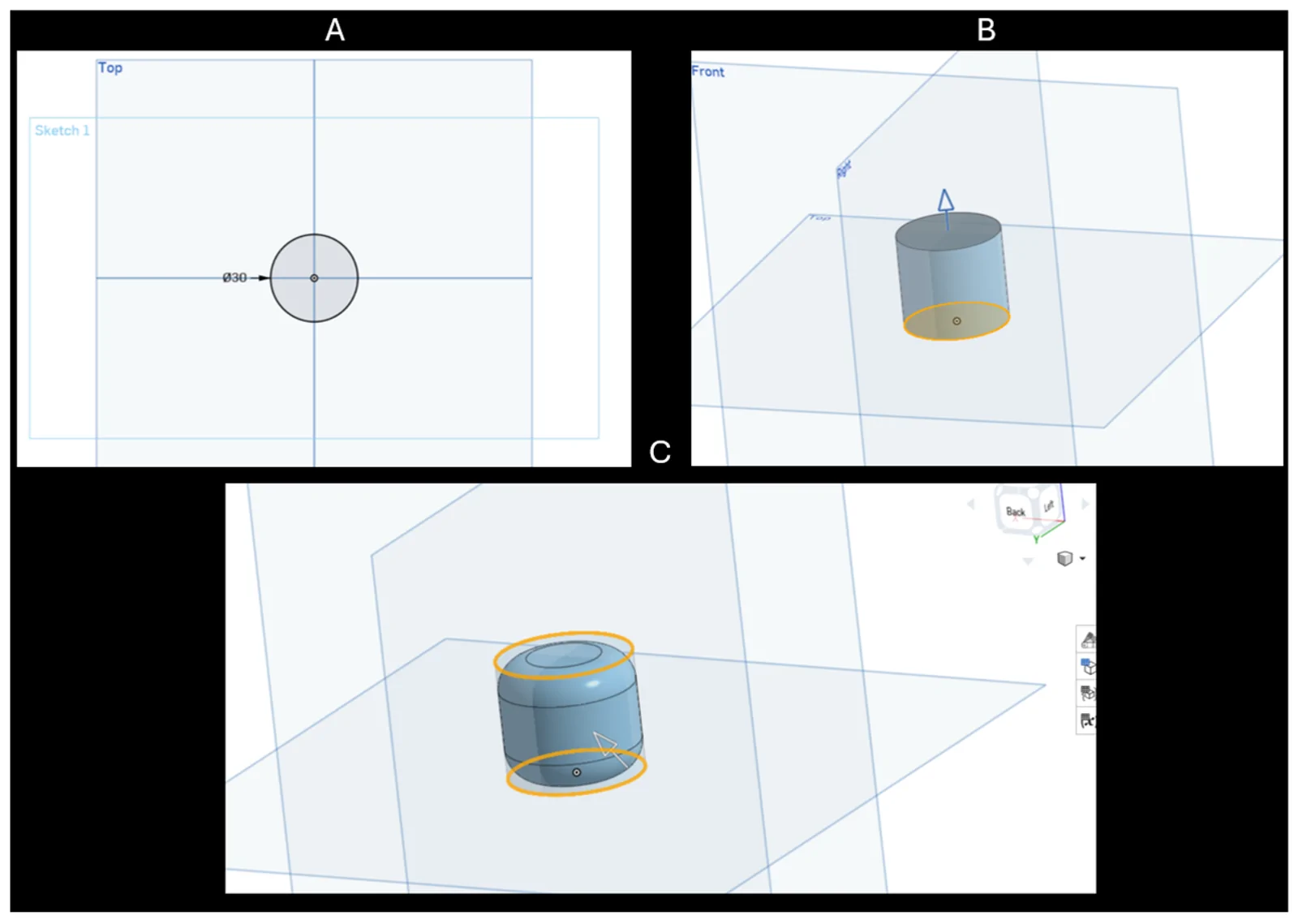
Modeling steps with Onshape online software. (**A**) Sketching in 2D. (**B**) Extruding sketch into a 3D digital object. (**C**) Modified shape of the digital object.

The very first step of manufacturing a 3D-printed implant is the designing of a Computer-Aided Design (CAD) model. The CADs are made in a modeling program (Figure 2), where the digital form of the implant can be modified until the desired size and shape is formed. There are several programs that are freeware or open source like FreeCAD, SketchUP, Onshape (Figure 2), Tinkercad, OpensCAD, or RepoCAD, as examples [28]. Then, these files should be converted into a Standard Tesselation Language (STL) file, which can be recognized by a slicer program [29,30].

**Figure 3:**
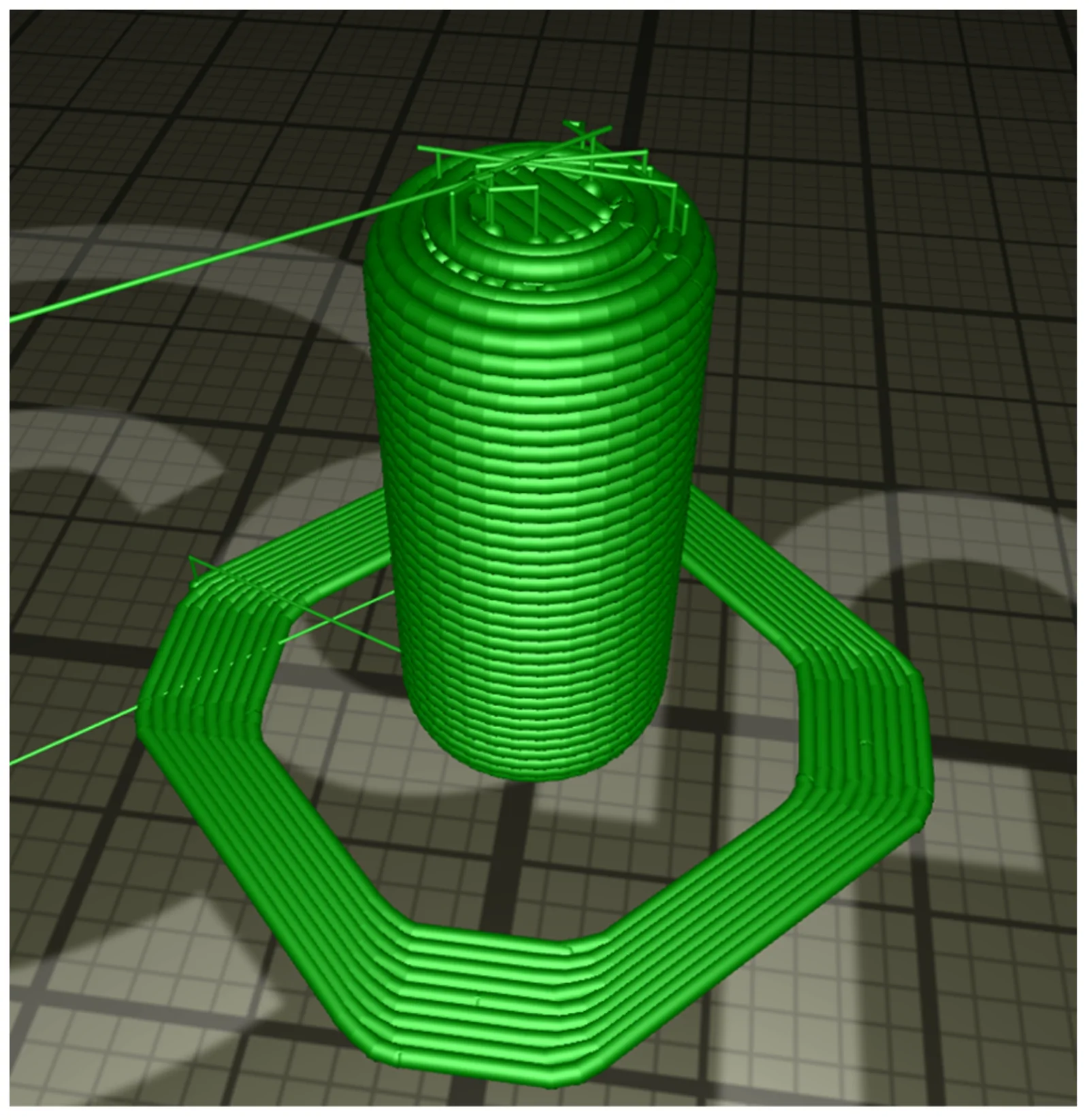
G-code of an object converted from .stl format in CraftWare slicer program.

In the slicer program, STL files are converted into G-codes (Figure 3), which contain the printing parameters mentioning operating temperature, layer height, distance between two layers, printer head movement speed, and other attributes, depending on the printer’s properties [29,30].

**Figure 4:**
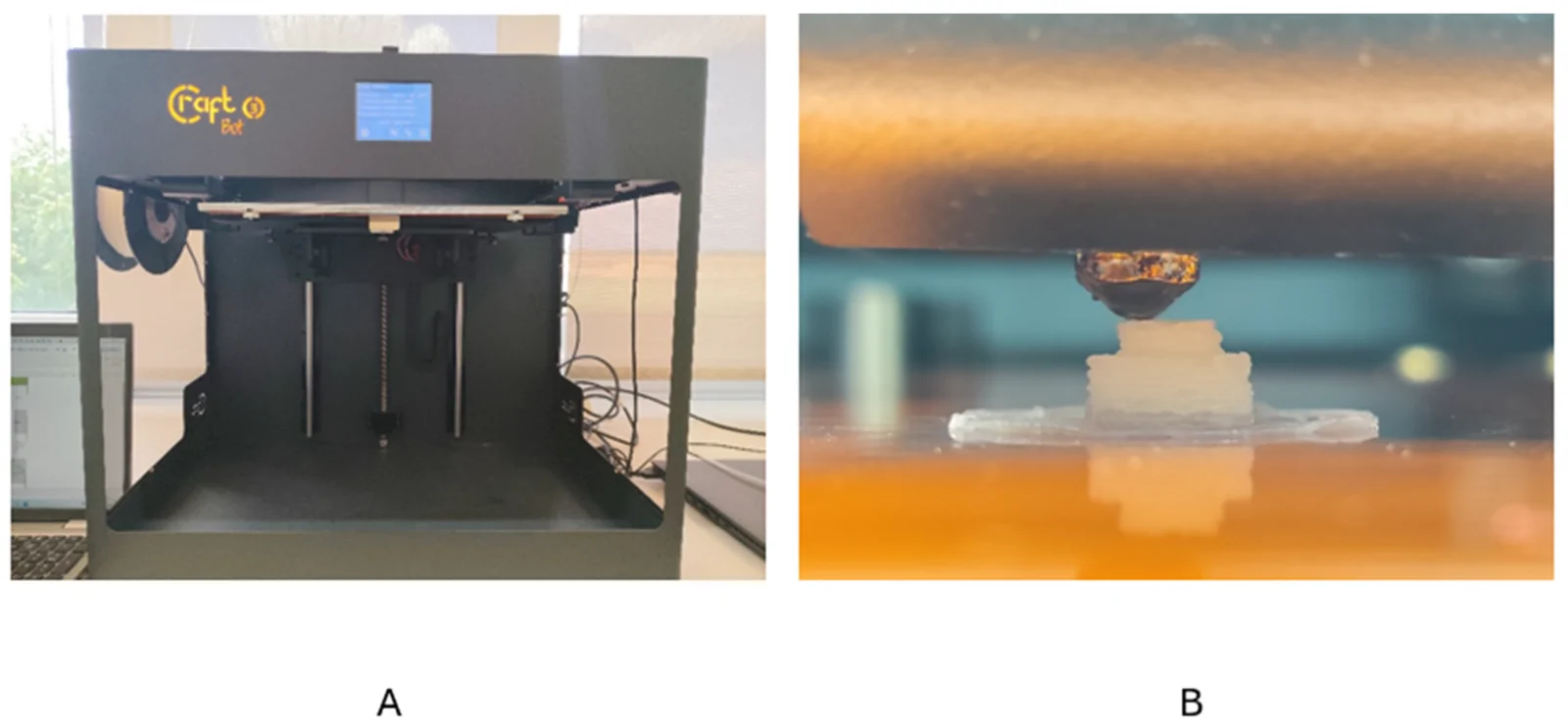
CraftBot FDM printer (**A**) and the printer head or nozzle (**B**).

Fused deposition modeling (Figure 4) uses thermoplastic polymer filaments as printing materials. A gear wheel drives the filament to the printer head’s heated nozzle, where the filament melts into a semi-solid state, and passes through to the printer bed, while the printer’s head moves horizontally, following the route determined by G-code to make a layer. Once the layer is completed, the bed or the printer head moves diagonally to get in position in order to apply a new layer. Optimally, the melted polymer solidifies in seconds after reaching the printer bed or the lower layer which is necessary for a stable base and smoother structure [31,32].

Critical printing parameters such as printer head diameter, layer height, infill density, infill pattern, printer head temperature, platform temperature, printing speed, and filament diameter highly influence the quality of the final object, which can be changed to achieve the desired result [27,33,34].

## 5. Polymers of HME-FDM-Printed Implants

Not all polymers are suitable for hot-melt extrusion or fused deposition modeling. Polymers that are used in HME and FDM processes, must have thermoplastic or thermo-softening properties to ensure smooth operation and avoid damaging the machines [35,36]. For pharmaceutical usage, especially in the case of implants, thermoplasticity is not enough for a polymer, it must be biocompatible, otherwise inflammation and immune rejection has a higher possibility to occur [37]. It is not obligatory to use only one kind of polymer at once. What is more, sometimes it can be advantageous if the polymers are mixed to improve each other’s stiffness and brittleness, or to achieve the desired effect and drug-release profile [38]. In this section, the HME-FDM-specific polymers used for implant manufacturing are described from the chosen articles. Some important parameters like molecular weight, viscosity, glass transition temperature (Tg), melting temperature (Tm), decomposition temperature (Td), or solubility were collected if they were mentioned in the article as they may represent an important critical material attribute (CMA); this can be found in Table 1. However, the authors found that biodegradability was mentioned, but the physical parameters were only partially or not mentioned in the chosen articles. This makes the reproducibility difficult. One critical aspect might be the different physical parameters resulting from the varying molecular weights of polymers, like in case of polycaprolactone (PCL), where the thermal degradation temperatures altered from the molecular weight PCL (Mw= 10,000) degrades at 309 °C, PCL (Mw = 45,000) at 332 °C and PCL (Mw = 80,000) at 336 °C [39].

## 6. Manufacturing of HME-FDM-Printed Implants

In this section, the manufactured implants are sub-grouped into different sections. The classification of devices proved to be a challenge since some of these IDDSs combine functionality and pharmaceutical purposes; therefore, the authors attempted to follow the FDA’s regulatory framework [48].

### 6.1. Subcutaneous Implant

Pediatric treatments often demand specific dosages considering age or even body weight. Three-dimensional printing gives the opportunity to personalize the size, shape, and, consequently, the dosage according to the patient’s needs. Daihom et al. formulated a gabapentin (GABA)-loaded long-acting medicinal implant for the treatment of pediatric neuropathy. They composed four formulations with GABA, using PCL and PEG. The GABA/PCL ratios were 10/80 *w*/*w*%, 20/70 *w*/*w*%, 30/60 *w*/*w*%, and 50/40 *w*/*w*%. For sustained release, PCL has already shown its effectiveness in different medical applications [49,50]. Each formulation contained 10 *w*/*w*% PEG3350 [43], which, in filaments, acts as a plasticizer and increases hydrophilicity [51]. In this research, their main objective was to develop an implant which has the potential to increase bioavailability as localized drug delivery and to reduce dosing to meet the patient’s need. Morphology and solid-state characterization, texture analysis, energy dispersive- X-ray (EDX) spectroscopy, and in vitro tests were also made for over 25 days. However, their main focus was to determine the effect of drug loading, infill densities, and surface area differences on the release profile, and to confirm the reliability of MeltPreps (melt preparations) in in vitro studies to accelerate development. In vitro drug release tests showed that higher drug loading resulted in higher initial release rates, increasing the availability of the drug; however, the higher drug-loaded implant’s release rate began to slow down with time compared to the lower loadings. The authors concluded that 3D printing was efficient in creating implants with the possibility of personalization due to the customizable release profiles [43].

Stewart et al. designed ibuprofen sodium-, ibuprofen acid-, and methylene blue-containing implants to compare each model drug’s behavior in the drug release profiles with differently designed implant structures with built-in drug release “windows”. These served as the surfaces where the drug could be liberated from the implant. They used PVA and PLA polymers to print hollow implant structures, then directly filled them with the model compounds. The combination of two types of PCL were also used as a coating material to obtain sustained drug release profiles. Their results showed that prolonged and sustained drug release can be acquired by a PCL coating, prolonging drug release up to 300 days while the surface area of drug release “windows” significantly influenced the drug release profiles. These results revealed that the design and the solubility of different APIs highly influence the drug-release rates, while the application of specific coatings placed on strategical sites of the implants provide the possibility of personalizing the release profiles according to the patient’s needs [44].

### 6.2. Drug-Eluting Implants

The use of drug-eluting implants can also be categorized based on the therapeutic purpose.

#### 6.2.1. Drug-Eluting Implant for Tissue Regeneration

For tissue regeneration monoclonal antibodies are already in focus [52]. However the effectiveness of such biological API highly depends on chemical and physical stability [53]. HME and FDM processes use relatively high temperatures during operation; therefore, thermosensitive APIs are rarely used directly in HME and FDM techniques. However, some polymers have the thermo–rheological properties to be potentially suitable for low-temperature operations that are also safe in terms of heat-sensitive APIs [54]. Carlier et al. used PLGA as a carrier for monoclonal antibody (mAb)-loaded implants. Filaments were made by HME, where PLGA, spray-dried mAb and PEG 2000 were blended previously. PEG 2000 was added as a plasticizer to decrease the glass transition temperature. Homogeneous dispersions were successfully reached both in filaments and in 3D-printed IDDS. Trehalose and L-leucine association (TRE-LEU) were used to protect and stabilize mAb against thermal degradation and, as a result, no fragmentation was observed, while dissolution tests showed a sustained-release profile [45].

#### 6.2.2. Drug-Eluting Surgical Implant

As a surgical implant, on-demand production of such devices may be necessary in urgent cases with customized dosage. A research group used an FDM technique with four different polymers, PLA, antibacterial PLA, PETG, and PMMA and diclofenac-sodium as a model drug for each, to print implantable samples. In this case, diclofenac-sodium was not embedded or extruded with the polymers, but rather they printed the bottom half of the hollow structure, filled it with diclofenac-sodium salt, put a pre-printed cover on top, then printed the top half upon that. Since the active pharmaceutical ingredient was not necessarily exposed to high heat, this method might be suitable for heat-sensitive APIs. In this early article, the focus was still on how to prevent the active ingredient from potential degradation through the FDM process, which the authors were able to provide, even though the API dissolution was within 24 h [42].

#### 6.2.3. Drug-Eluting Implant for Sustained Blood Concentration

Yang et al. reported that a PCL matrix releases the embedded APIs at slow rates, so they proposed mixing PCL with chitosan to improve drug release. As a model drug, they used ibuprofen and extruded their own filament by HME, then successfully applied the filament to their FDM printer. Chitosan acted as a plasticizer in the filament and reduced the complex viscosity. In vitro results showed that the drug content crucially influenced the drug release. When the ibuprofen content was increased from 4 wt% to 20 wt%, the overall drug release also increased from 28% to 100%. Drug release was influenced by the chitosan too. When they changed from 4 wt% to 20 wt% chitosan, the release of the drug also increased from 55% to 100%. The reason for the changes in both cases was the formation of more release channels. As they concluded their results, the proper amount of chitosan could form enough release channels for the ideal performance, while acting as a plasticizer and improving the printability of the PCL filament [41].

### 6.3. Intrauterin Implant

Uterine cancer is one of the most common cancers affecting women. Varan et al. proposed using paclitaxel and carboplatin separately in two different filaments for 3D-printed implantable intra-uterine device (IUD). They encapsulated both drugs in different cyclodextrin derivatives with different ratios, where they found out the methyl-β cyclodextrin (MβCD) was the most efficient in both cases. The main structure of their filament was polycaprolactone. Differential scanning calorimetry (DSC) results showed that the different formulations’ melting peaks were close to 60 °C, similar to pure PCL. This indicates a possibly safe material choice for filament manufacturing, avoiding high temperatures when thermosensitive drugs and drug complexes are used. Drug release tests also confirmed the prolonged drug release in case of paclitaxel–cyclodextrin complex, where in 10 days, only 15% of the total drug amount was released. This is suitable for prolonged release profiles while, in case of carboplatin–cyclodextrin complex, the overall release was faster. In the case of paclitaxel, carboplatin was released independently from the PCL degradation rate. These interesting results suggest that the choice of drugs in anti-cancer therapies is far more significant, considering drug release profiles in personalized treatment strategies [39].

### 6.4. Scaffold Implant

Mei et al., in an effort to avoid these side effects and directly target the tumor cells, designed an anti-tumor implantable porous scaffold, using PLA and methotrexate (MTX) which were extruded into a filament. The targeting of tumor cells was based on the possibility of using the patient’s computed tomography (CT) and magnetic resonance imaging (MRI) to design and personalize the 3D model according to the tumor’s structure. An FDM printer was used to print the scaffolds, and these scaffolds were placed on tumors inside different mice groups. In comparison to the injection- and implant-treated animals, the mice that were treated with injections had a lower organ coefficient on the spleen in opposition to the scaffold implant-treated animals, while the scaffolds efficiently inhibited the growth of the tumor. In vitro test results also showed that MTX was released along with PLA degradation and the growth of tumor cells was successfully and significantly inhibited after 24 h. After 120 h, a 95% inhibition rate of tumor cells was measured, while for the growth of normal cells, the effect was negligible [46].

### 6.5. Drug-Eluting Functional Implants

#### 6.5.1. Drug-Eluting Cardiovascular Prosthesis

In relation to cardiovascular diseases, many types of implants have been designed such as stents, grafts, and valvular prosthesis. The implantation of these devices has the risk of developing infections, or, in some cases, blood clots can form after operations. Martin et al. printed a vascular graft from TPU and an antibiotic, rifampicin, was embedded within it as a prophylactic agent. The same methodology was used with another drug, dipyridamole, which is an antiplatelet drug to prevent cardiovascular device-related thrombosis. In vitro release tests were performed with the two separate drug-loaded implantable devices. The rifampicin-containing device showed that it was capable of sustained release for more than 30 days, which might predict a potential and successful prevention from infections after surgery in the time range when most of the infections occur. In the case of the dipyridamole-containing device, sustained release was observed for 55 days; however, the test result predicted a longer period for the sustained release profile. Their conclusion was that it is possible to produce filaments with HME and FDM to print a cardiovascular device that can possibly prevent infections and blood clots forming after surgery [40].

#### 6.5.2. Drug-Eluting Fixation Implant

Implantable devices in orthopedic surgery are commonly used for multiple purposes. A research group fabricated FDM-printed patient-specific fixation implants which allow local drug delivery. Gentamicin and methotrexate were impregnated into the PLA implants. The devices successfully showed both antibacterial and chemotherapeutic effects; however, they negatively influenced the overall strength of the implants [47].

### 6.6. Formulation Strategy

Following revision of the chosen articles, the authors concluded that for the development of IDDSs, several drug-loading techniques have been found that allow incorporation of thermo-sensitive APIs, but some of these require specific circumstances before, during, or after the procedure. While the main focus is on polymer types and formulations, no article has discussed API behavior comprehensively with these formulations. The above-mentioned studies focusing on solving 3D-printed IDDS-related issues from the aspect of chosen API, the formulation strategy, and the innovative approach can be found in Table 2.

Regarding the reviewed articles, there would be great opportunity for implant manufacturing and a great demand from several aspects as these techniques do not allow the possibility of personalization. The technology is already available and has been extensively used in the last decade, but some aspects are still missing for better quality and reproducibility.

For a better understanding regarding our research group’s recent work examining quality by design (QbD) possibilities for better quality outcomes in HME-FDM, see Table 3. The critical process attributes (CPPs) were collected for both HME and FDM processes regarding the collected articles and the provided data. In the case of HME, the extrusion temperature, extruder screw speed, and die diameter was revised. In the case of FDM, the layer height, infill density and pattern, print head diameter and temperature, platform temperature, and the printing speed were collected [27].

## 7. Characterization and Evaluation

Evaluating a filament and the finished 3D-printed implant is crucial for determining the safe and effective applicability of the final product. Structural integrity, biocompatibility, chemical stability, and stable drug-release profiles are demanded in all cases, and these can be examined using several methods applied to IDDSs.

The characterization is sub-grouped regarding the system of Repka et al. [19].

### 7.1. Physico-Chemical Characterization

The physico-chemical characterization is made possible by using powder X-ray diffraction (PXRD), Fourier transform infrared spectroscopy (FTIR), NIR, or nuclear magnetic resonance spectroscopy (NMR).

#### 7.1.1. PXRD

The scattering of X-rays from atoms in a crystalline material creates diffraction patterns that reveal atomic arrangements in samples. The result is a diffractogram with diffraction patterns which consist of peaks [55]. This characterization technique can determine if the API is in an amorphous or crystalline form in the polymer. By varying humidity and temperature values, it also provides information about the stability of the final product [19]. In the case of the polycaprolactone–chitosan-based implant, the crystallization temperatures of PCL and ibuprofen were determined with this method. It was also confirmed that, after the extrusion and printing process, PCL retained its semi-crystalline state and ibuprofen remained in crystalline form. It also provided valuable information about composite filaments, revealing that the diffraction peaks of the polymer decreased as the drug-loading of the filament increased [41].

#### 7.1.2. FTIR Spectroscopy

FTIR is used to identify molecules or molecular groups by their vibrational properties [56]. In terms of 3D-printed implants, it helps to identify possible chemical changes that occur during the manufacturing process and chemical interactions between component materials [57,58,59]. Although chemical changes between substances are unwanted in most of the cases, Yang et al. mentioned that FTIR analysis revealed that hydrogen bonds were formed between the hydroxyl group of PCL and the hydroxyl group of ibuprofen which improved the interfacial compatibility, while other chemical bonds were not formed [41]. Varan et al. compared blank, drug-loaded PCL filaments, and PCL granulates to determine if the extrusion process or drug-loading affected the melting peaks through the process; however, the similar melting peaks suggest that neither the process nor the drug-loading influenced the chemical structure significantly enough to change the melting properties of the filament [39].

### 7.2. Thermal Characterization

Thermal characterization is important in the manufacturing process, and is widely used for pre-, intermediate, and final analytical testing. It plays an important role as the HME-FDM process applies high heat on the components, which may result in thermal degradation. Thermal characterization techniques are also effective in the determination of glass-transition temperatures and melting temperatures [54]. It is crucial to perform these experiments as HME-FDM can expose drugs and excipients to heat and shear must be kept in mind in the case of peptides, antibiotics, and thermolabile small molecules.

#### 7.2.1. Thermogravimetric Analysis (TG/TGA)

Thermogravimetric analysis is used to investigate thermal decomposition and to assess relative thermal stabilities [60]. In the case of 3D-printed implants, it provides valuable information about the different thermal degradation behaviors of pure polymer, polymer–polymer, polymer–excipient, and drug-loaded polymer mixtures [41,61,62]. For this analysis, a small sample, usually less than 10 mg, is more than enough. From the results, it is possible to determine the safe operation temperatures for filament extrusion and 3D printing, thereby avoiding critical API loss and other unwanted thermochemical reactions and instabilities [45,57,58]. For the cardiovascular prosthesis, TGA was sufficient in the determination of rifampicin stability at the extrusion temperatures, while the analysis also revealed that the rifampicin-containing TPU filaments showed higher degradation temperatures compared to pure TPU [40].

#### 7.2.2. Differential Scanning Calorimetry (DSC)

Differential scanning calorimetry is a thermal analysis technique that is also applicable to various kinds of materials such as pharmaceuticals including implants, and polymers also used for 3D printing [63]. With DSC, polymer raw materials, filaments, and even polymer–excipient–drug mixtures can be monitored to describe the glass transition temperatures and changes as other materials are blended together. This both helps to determine the necessary operation temperatures during heat transfer processes, and also if the components dissolve into each other at such temperatures or stay in a crystalline form [43,45,59,64]. In most cases, TG and DSC analysis are evaluated together for a more complex and precise result [41,58].

### 7.3. Microscopic Characterization

Microscopic characterization is usually performed by scanning electron microscopy (SEM), micro-computed tomography (micro-CT or μCT), X-ray-computed tomography (XRCT), hot-stage microscopy (HSM), or polarized light microscopy (PLM).

#### 7.3.1. SEM

Scanning electron microscopy provides a surface evaluation method for larger (200 mm or in some cases even larger) samples with up to 1,000,000× magnification and 1 nm resolution opportunity [65]. The usage of SEM helps to characterize the structural integrity and morphology of implants, revealing possible defects or changes that occur during or after printing, and also after going through several tests such as in vitro release studies [41,64]. SEM images are able to show possible drug aggregates within the extruded filaments or printed implants, from which we can also obtain valuable information about homogeneity which can affect the drug release profile [57,59]. The other crucial factor that must be examined is proper layer adhesion, which needs to be intact in order to ensure durability, mechanical robustness, tissue integration, and biocompatibility [43].

#### 7.3.2. MicroCT

MicroCT has the ability to visualize and quantify porosity or defects in IDDS. This density measurement method allows the detection of inclusions, unconsolidated powder, and open porosity at the surface with visualization and part mass density. Part density is usually measured by the Archimedes method; however, in additive manufacturing, the rough surfaces which trap gas, infiltrated water through the pores, and the unconsolidated powder can conceal the presence of porosity in the density measurement. Rough surfaces also influence the microCT measurements; however, proper determination of the sample edge and regular calibration of the instrument can reduce the error margin [66].

#### 7.3.3. Hot-Stage Microscopy (HSM)

HSM provides information about thermal events visually, which helps describe the polymer–API interaction in a molten state, such as the dissolution and recrystallization effects of temperature [19].

#### 7.3.4. Polarized Light Microscopy (PLM)

PLM enables the observation and the visualization of state transitions by applying heat. With an optical microscope to which a camera is attached, video documentation can be conducted saving time, money, and other resources without repeating the experiment [35].

### 7.4. Pharmacopoeial Testing

Regarding the pharmacopoeial testing of 3D-printed IDDSs, in vivo and in vitro tests are well-described for conventional manufacturing of these systems. For this reason, the authors only focus on the most important aspects [20].

#### 7.4.1. In Vitro Dissolution Testing

In vitro drug release tests help to determine drug release profiles of IDDS, where the release medium imitates the tissue environment. Samples are taken over time to predict the implant’s drug release kinetic behavior once it is implanted [67]. Significant differences were shown between polymers in drug release in different polymer–drug pairings, which provides information to select the proper polymer–drug combinations, considering the advantages and disadvantages of formulations depending on the future use [38,42,45,67]. Studies have shown that the solubility of the drug in the release medium also affects the release kinetics. How the drug behaves at different amounts of drug loadings must be taken into account to avoid burst effects and overdosing as the aim of implants is to ensure a well-defined, sustained, and prolonged release profile [44,68]. In the case of 3D-printed IDDSs, it is not only the change in polymer–drug ratio and the type of the polymer influences drug release, but also implant design is important, as surface area and structural modules can be changed with different shape designs. This could be a possible method for personalized release profiles [62]. In vitro dissolution testing is an important question and can provide a good prediction of the quality of the manufactured product. In Table 4, the authors collected the used API or model compound, the used polymer, the dissolution test circumstances, and the results for a better understanding of where we stand right now in implant manufacturing by HME-FDM.

#### 7.4.2. Shelf Life

The shelf life of different products still has not been determined or tested among the mentioned IDDSs, or if sterilization methods, where applied, were effective enough to store these devices for a longer period of time. There is also the question of how much storage time would be ideal for such devices [39,40,41,42,43,44,45,46].

### 7.5. Biocompatibility

The biocompatibility measurement of implantable devices is essential. The implantation of a foreign object triggers a host response, including an immune response in the body. Host response includes inflammation at the implant site, tissue remodeling, and proliferation. During an immune response, the innate and adaptive immune systems are the definitive mediators, where the innate immune response is responsible for the nonspecific inflammatory response. The innate immune system also triggers the adaptive immune response where specific antigens are involved. These antigens may react to dissolving or degrading implant components, causing rejection. To avoid a host response, the testing of such devices involves device design, in vitro study of cytotoxicity, in vivo study of device performance using animal models, genotoxicity and cellular response to the materials or devices, and the final translation to the clinical testing [69]. For biocompatibility and cytotoxicity testing, several assays such as LDH test or real-time electronic sensing assay (RT-CES) may be used; however, the most widely used method is the rapid colorimetric 3-(4,5-dimethylthyazol-2-yl)-2,5-diphenyl-2H-tetrazolium bromide dye (MTT) cytotoxicity test, where the parameters are determined by the International Organization for Standardization (ISO) including exposure period, type of positive control, and the number of cells in the dishes before and after exposure to the material [42,70].

The surgical implant’s MTT assay proved that the different samples including PLA, antibacterial PLA, PETG, and PMMA are cytocompatible even after extrusion and printing [42]. The cardiovascular graft containing rifampicin was tested with a different method, by culturing HUVEGs onto the grafts after sterilization. Samples were observed for 72 h, and the results showed that the cells attached to the surface of the sample and only a few cell deaths were observed. A monolayer of HUVEGs also appeared on the surface and the graft samples proved to be non-cytotoxic [40].

#### 7.5.1. In Vivo Tests

In vivo tests are often conducted on animals such as mice. These tests usually last for a couple of months to ensure reliable results. They are necessary to establish biocompatibility and to investigate further the polymer degradation mechanism or the tissue reaction by explantation of the implant and removing the surrounding tissues at the end of the experiment for quantitative and qualitative testing. Valuable information about tissue–material interactions such as cell response, tissue formation behavior, vascularization of newly formed tissues, and host response behavior can be evaluated before human clinical trial phases [71].

#### 7.5.2. Sterilization

Just to mention the sterilization methods, HME-FDM 3D-printed implants still need more thorough study to examine possibly applicable processes to meet the determined demands and quality requirements of implants. This is important as they may affect the polymer molecular weight, drug potency, impurity profile, crystallinity, mechanical properties, surface wettability, residual moisture, and release kinetics [72]. However, some efforts to test different techniques, such as γ-sterilization, have been used by some groups; the method was conducted according to the proper ISO determined parameters [73]. PLA and heat-treatable PLA (PLA with crystallization compound) went through autoclave sterilization in case of an FDM-printed orthopedic cutting guide. Although this procedure was not applied to an implant, it did not cause any substantial negative effect on the dimensional functionality. Theoretically, in the case of PLA implants where a post-print drug loading process is applied, this might be a strategy worth considering for further investigation [74]. Other research groups performed several sterilization methods on different polymers, including PLA and PETG. The applied sterilization methods such as autoclave (with higher and lower heat exposure), ethylene oxide, and hydrogen peroxide plasma proved to be efficient in destroying microorganisms. High-temperature autoclave sterilization significantly affected the structure of the polymer, while in lower temperature environments, less deformation occurred. Gas sterilization eliminates deformation issues; however, it could have a significant impact on chemical structure [72]. In another study, they used 70% (υ/υ) ethanol for microbial reduction in which the implant was immersed for 12 h in a laminar air flow cabinet; however, the possible effects of this decontamination were not examined further in detail [42]. It will be crucial to place greater emphasis on the possible sterilization of HME-FDM manufactured implants.

### 7.6. Contact Angle Measurement

Wettability determines crucial biological and chemical processes. The contact angle is defined as the angle between the tangent and the three phase point and the solid surface. The lower the contact angle, the better the wetting properties of the solid object as hydrophilicity increases [75]. Surfaces with contact angles < 90° are considered hydrophilic, while contact angles > 90° are considered hydrophobic [76]. The implantable devices drug delivery rates are also highly determined by surface wettability, which is also determined by polymer structures [77]. Arany et al. compared the contact angle values of PLA and antibacterial PLA filaments, where the contact angle value of antibacterial PLA was nearly three times smaller than PLA, which was probably caused by the antibacterial nanoparticles [42]. M’Bengue et al. and other studies showed that some sterilization methods like gamma sterilization could influence contact angle values as after irradiation of TPU. Here, hydrophobicity decreased, which indicates that after sterilization, contact angle measurements should be repeated considering the significance of wettability of implants [73,78].

## 8. Translation and Regulation

There are already some opportunities for 3D printing technology and its industrial application, like the Triastek™ Melt extrusion deposition = MED^®^ manufacturing, which is extrusion-based, allows continuous manufacturing, and is FDA approved [79]. In the author’s opinion, in the case of HME-FDM, the main concept would be to manufacture personalized medications, not only in dose but in design.

The regulatory environment is in development by agencies around the world to ensure patient safety while allowing innovative approaches [80]. The most advanced regulatory approach currently is Framework for Regulatory Advanced Manufacturing Evaluation (FRAME) which is under development by the FDA. FRAME’s main focus is on artificial intelligence, end-to-end continuous manufacturing, distributed manufacturing, and distributed manufacturing at non-traditional host sites [79]. HME is considered as a continuous manufacturing method, while 3D printing including FDM is a distributed manufacturing method [79,81]. This direction is forward-looking and will hopefully contribute to the regulatory acceptance of HME-FDM products.

## 9. Limitations

As with every technology, HME-FDM 3D printing has its own limitations. Concerns have been raised by professionals in the healthcare industry about medication safety and quality aspects mentioning stability, quality control, dose accuracy, shelf life and the identifications of drug products [82]. Other mentioned limitations include relatively low productivity, high costs, and issues about on-demand production and delivery [83]. However, the major limitation of the technique is the relatively low printing efficiency. Others have also raised issues about surface quality and mechanical strength of the final object [84,85].

There are some limitations, but we must keep in mind that every technology has at least some. In the author’s opinion, 3D printing, such as HME-FDM, including the QbD principles and the ability to manufacture reproducible and determined quality products, could become an effective tool for personalized drug product manufacturing. Personalized drug products, including drug-loaded implants, will be much more widespread in general healthcare, considering the need to vary dosage based on age and weight, and the possibility for 3D printing to influence release profiles and dosage by customized shapes and size [86].

## 10. Conclusions

Implantable drug delivery systems have demonstrated their significance in long-term treatments and therapies; however, it can be challenging to achieve the personalization of dosages and anatomical compatibility through traditional manufacturing processes. Three-dimensional printing could possibly provide an easier solution to these difficulties, considering the results research achieved to date, as well as polymers, APIs, characterization, and evaluation methods that are already used in the pharmaceutical industry. Numerous research groups are also working on the possible application of 3D printers and computer-aided programs that are still improving, but there are still some gaps between the research and the final applicable product. HME-FDM can be a potential manufacturing process for IDDSs, where the applicable materials, including some thermoplastic polymers, have the background for the use of medical purposes. In general, the characterization and evaluation methods are ready for the commercially available IDDSs, but some personalization could be included, and the quality and reproducibility should be provided in harmony with the QbD guidelines. The regulations and manufacturing standards for 3D-printed drug delivery systems are still under development.

Overall, 3D-printed IDDSs have the potential to provide another kind of safe, more patient-friendly, and personalized medication form in various treatments and therapies for both children and adults, but more research should be performed to achieve this goal.

## Figures and Tables

**Table 1:** Biocompatible polymers used in HME-FDM for implant manufacturing. The parameters, solubility, and biodegrability statements are based on the revised articles.

Biocompatible Polymers Demonstrated in HME and FDM	Molecular Weight (Mw), Viscosity, Tg, Tm, Td	Solubility	Biodegradability	References
Thermoplastic polyurethane (TPU)	Mw: Not mentioned Tg: Not mentioned Tm: Not mentioned Td: Not mentioned Viscosity: Not mentioned	Water-insoluble	Non-biodegradable	[40]
Chitosan (CS)	Mw: 1000 kDA Tg: Not mentioned Tm: Not mentioned Td: Not mentioned Viscosity: Not mentioned	Water-soluble	Biodegradable	[41]
Poly (methyl methacrylate) (PMMA)	Mw: Not mentioned Tg: 105 °C Tm: Not mentioned Td: 340 °C Viscosity: Not mentioned	Water-insoluble	Non-biodegradable	[42]
Polycaprolactone (PCL)	Mw: 550 Da; 10,000 Da; 45,000 Da; 50,000 Da; 80,000 Da Tg: 58.66 °C; 58.7 °C Tm: 58.66 °C; 58.7 °C Td: 309 °C; 332 °C; 336 °C (in case of Mw = 10,000 Da; 45,000 Da; 80,000 Da) Viscosity: Not mentioned	Water-insoluble	Biodegradable	[41,43,44]
Polyethylene glycol (PEG)	Mw: 3350 Da; 1000 Da; 2000 Da Tg: 59.55 °C Tm: 59.55 °C Td: ~200 °C Viscosity: Not mentioned	Water-soluble	Biodegradable	[43,44,45]
Polyethylene terephthalate glycol (PETG)	Mw: Not mentioned Tg: 80 °C Tm: Not mentioned Td: 400 °C Viscosity: Not mentioned	Water-insoluble	Non-biodegradable	[42]
Polylactic acid (PLA)	Mw: 500,000 Da Tg: 55–60 °C Tm: Not mentioned Td: 280 °C Viscosity: Not mentioned	Water-insoluble	Biodegradable	[42,44,46,47]
Poly lactic-co-glycolic acid (PLGA)	Mw: 7000–17,000 Da Tg: 38.0 ± 0.7 °C Tm: Not mentioned Td: Not mentioned Viscosity: Not mentioned	Water-insoluble	Biodegradable	[45]
Polyvinyl alcohol (PVA)	Mw: Not mentioned Tg: Not mentioned Tm: Not mentioned Td: Not mentioned Viscosity: Not mentioned	Water-soluble	Biodegradable	[44]

**Table 2:** IDDSs and innovative approach in their research.

Implant Type	API(s)/Model Compound	Formulation Strategy	Innovative Approach/Main Focus of the Article	Reference
Subcutaneous implant	Gabapentin	Drug-blend technique through HME	Solving bioavailability issues observed in oral administration Reduced dosing	[43]
Subcutaneous implant	Ibuprofen sodium, ibuprofen acid, methylene blue	Hollow implant fill with API	Testing the significance of surface area, drug solubility, and polymer coatings for sustained release considering parameters for personalized treatment	[44]
Drug-eluting implant for tissue regeneration	Monoclonal antibody	Drug-blend technique with HME, API encapsulation for stability	First thermosensitive mAb-loaded implant manufacturing with FDM ~25 days of in vitro test	[45]
Drug-eluting surgical implant	Diclofenac sodium	Hollow implant fill with API	In vitro drug release tests Separate filament extrusion and drug loading to avoid heat degradation	[42]
Drug-eluting implant for systemic sustained drug concentration	Ibuprofen	Drug-blend technique with chitosan through HME, chitosan forming release channels through dissolution	Improving printability and influencing drug release rates by adding chitosan to the filament as plasticizer	[41]
Chemotherapeutic intrauterine implant	Paclitaxel, carboplatin	Drug-blend technique with HME	Personalized treatment for uterine cancer with local drug delivery	[39]
Chemotherapeutic scaffold implant	Methotrexate	Drug-blend technique with HME using porous polymer	Personalizing treatment with the use of CT and MRI images for 3D modeling Controlled drug release by PLA degradation and efficacy studies. Reduced side effects compared to traditional administration	[46]
Drug-eluting cardiovascular graft	Dipyridamole, rifampicin	Drug-blend technique with HME	Prevention of cardiovascular prosthesis surgery-related infections and blood clot formation	[40]
Drug-eluting fixation implant	Gentamicin, methotrexate	API impregnation into printed polymer implant	Embedding antibiotics and chemotherapeutics into fixation implants	[47]

**Table 3:** In the case of the revised articles, some CPPs were collected for HME-FDM manufacturing.

Implant	HME Parameters	FDM Parameters	Reference
Gabapentin-loaded implant	Extrusion temperature zones (°C): 40; 50; 60; 80; 80; 80; 80; 70; 60 Extrusion speed (rpm): 50 Die diameter (mm): 1.75	Layer height (mm): 2 Infill density (%): 25; 50; 100 Infill pattern: linear Print head diameter (mm): 0.4 Print head temperature (°C): 110 Platform temperature (°C): 30 Printing speed (mm/s): 5 Filament diameter (mm): 1.75	[43]
Prolonged drug-delivery implant	Extrusion temperature zones (°C): 170–190 Extrusion speed (rpm): 5 Die diameter (mm): Not mentioned	Layer height (mm): 0.2 Infill density (%): Not mentioned Infill pattern: Not mentioned Print head diameter (mm): 0.4 Print head temperature (°C): 205 Platform temperature (°C): 85 Printing speed (mm/s): 70	[44]
Monoclonal Antibody-loaded implant	Extrusion temperature zones (°C): 20; 40; 80; 90; 90; 90; 85; 75 Extrusion speed (rpm): 40 Die diameter (mm): 1.6	Layer height (mm): 0.1; 0.3 Infill density (%): 100 Infill pattern: Not mentioned Print head diameter (mm): 0.5 Print head temperature (°C): 105 Platform temperature (°C): Printing speed (mm/s): 1 (first layer) 10 (layers above) Filament diameter (mm): Not mentioned	[45]
Surgical implant	Extrusion temperature zones (°C): Not mentioned Extrusion speed (rpm): Not mentioned Die diameter (mm): Not mentioned	Layer height (mm): 0.2 Infill density (%): 0; 5; 10; 15 Infill pattern: cubic Print head diameter (mm): 0.4 Print head temperature (°C): PLA: 215 PETG: 250 PMMA: 270 Platform temperature (°C): PLA: 60 PETG: 90 PMMA: 110 Printing speed (mm/s): Not mentioned Filament diameter (mm): 1.75	[42]
Implant for sustained blood concentration	Extrusion temperature zones (°C): 100; 105; 105; 100 Extrusion speed (rpm): 220–240 Die diameter (mm): Not mentioned	Layer height (mm): 0.2 Infill density (%): 20 Infill pattern: Not Mentioned Print head diameter (mm): 0.8 Print head temperature (°C): 120 Platform temperature (°C): 0 Printing speed (mm/s): 60 Filament diameter (mm): 1.75	[41]
Anti-tumor intra uterine implant	Extrusion temperature zones (°C): 75; 80; 80; 75 Extrusion speed (rpm): 2 Die diameter (mm): Not mentioned	Layer height (mm): Not mentioned Infill density (%): Not mentioned Infill pattern: Not mentioned Print head diameter (mm): Not mentioned Print head temperature (°C): Not mentioned Platform temperature (°C): Not mentioned Printing speed (mm/s): Not mentioned Filament diameter (mm): 1.75	[39]
Anti-tumor scaffold implant	Extrusion temperature zones (°C): 220 Extrusion speed (rpm): 45 Die diameter (mm): Not mentioned	Layer height (mm): Not mentioned Infill density (%): Not mentioned Infill pattern: Not mentioned Print head diameter (mm): 0.4 Print head temperature (°C): 210 Platform temperature (°C): Not mentioned Printing speed (mm/s): Not mentioned Filament diameter (mm): 1.75	[46]
Drug-loaded cardiovascular prosthesis	Extrusion temperature zones (°C): 170–200 Extrusion speed (rpm): 35 Die diameter (mm): Not mentioned	Layer height (mm): 0.1 Infill density (%): Not mentioned Infill pattern: Not mentioned Print head diameter (mm): 0.4 Print head temperature (°C): 215–220 Platform temperature (°C): Not mentioned Printing speed (mm/s): Not mentioned Filament diameter (mm): 2.85	[40]
Drug-eluting fixation implant	Extrusion temperature zones (°C): 170 °C Extrusion speed (rpm): Not mentioned Die diameter (mm): Not mentioned	Layer height (mm): Not mentioned Infill density (%): 25; 50; 75; 100 Infill pattern: honeycomb Print head diameter (mm): Not mentioned Print head temperature (°C): 215 Platform temperature (°C): Not mentioned Printing speed (mm/s): 8–12 Filament diameter (mm): 1.75	[47]

**Table 4:** Summaries of the used API or model compound, the used polymers, and the dissolution test circumstances and results of the reviewed articles.

API(s)/Model Compounds	Polymers	Dissolution Test in Agitated Vessel Models (Circumstances)	Dissolution Test (Results)	References
Gabapentin	PCL	Used MeltPreps, Medium: 20 mL of phosphate-buffered saline (PBS, pH 7.4), Incubator temperature: 37 °C Continuous agitation at 100 rpm. Sample volume: 1 mL (volume replaced with new buffer)	0–5 days: rapid release (30%) 5–15 days: steady release rate 33–35% 15–30 days: release stabilized at 38–42% by day 25 Effect of drug loading: higher loading exhibited faster initial release (by day 5). Drug content: 30%*w*/*w*: 35% cumulative release 50%*w*/*w*: 40% cumulative release Between 5 and 15 days both reached steady release at 50–55%.	[43]
mAb	PLGA	~200 mg 3D-printed IDDS Medium: 5 mL of PBS (0.2 M, pH 7.0) Incubator temperature: 37 °C Stirring at 600 rpm Sample volume: 5 mL	0–3 weeks: hydration occurred, pH remained constant, cumulative release <10% 3–7 weeks: degradation increased, pH decreased to ~6.3 cumulative release ~40% (64% mass loss) 7–15 weeks: No further degradation reported (week 10), polymer mass remained stable, pH increased from 6.7 (week 8) to 7 (week 15)	[45]
Diclofenac sodium	PLA, PETG, PMMA	15 implant samples Medium: 900 mL phosphate buffer (1.0 M, pH 7.4) Rotation speed set at 100 rpm Incubation temperature 37 °C Sample volume: 3 mL	0–2 h: dissolved API amount varied from 16 to 97%. In two cases the dissolution reached the plateau phase. 2–24 h: The dissolved API amount varied (~50% to ~99%) PETG and PMMA samples showed the highest values (99.49% and 99.48%) The lowest release rate was shown by a PLA sample (51.26%)	[42]
Methylene blue, ibuprofen sodium, ibuprofen acid	PLA, PVA, PCL (applied as a coating)	Implants 2.5 × 40.0 × 1.7 mm (A, B, C, D, E) with different PVA “window” size Implants loaded with 3 different drugs. Medium: 500 mL of PBS (also contained 0.05% sodium aside for ibuprofen sodium, and acid) Incubation temperature: 37 °C Shaken at 40 rpm. Sample volume: 0.5 mL	Implant A: 100% drug release within 24 h made entirely from PVA Implant B: “window”: one 1 × 38.0 mm: ~100% release at day 6 days Implant C: “window”: eight 1.0 × 1.0 mm: ~100% release at day 6 Implant D: “window”: two 1.0 × 1.0 mm: ~100% release at day 18 Implant E: “window”: one 1.0 × 1.0 mm: ~100% release at day 24 (Decreasing the surface area for drug release “windows” resulted in a prolonged release profile) PCL coatings provided extended-release profile for implant B (>300 days)	[44]
Paclitaxel, Carboplatin	PCL	1 cm samples from filaments (a: paclitaxel containing and b: carboplatin containing) Medium: ultrapure water, PBS buffer (pH 7.4), Citrat buffer (pH 5.5) containing Tween 80% 0.1 (*v*/*v*) Drug release was measured by HPLC Incubation temperature: 37 °C Samples kept in shaker water bath (was not detailed further)	No release was seen in the first 12 h *Day 1*: Paclitaxel: Water, PBS, Citrate: ~23% cumulative release Carboplatin: Water PBS, Citrate: ~0% cumulative release *Day 2*: Paclitaxel: Water, Citrate: ~3% cumulative release, PBS: ~5% cumulative release Carboplatin: Water: ~10% cumulative release, PBS: ~15% cumulative release, Citrate: ~0% cumulative release *Day 5:* Paclitaxel: Water: ~8% cumulative release, PBS: ~9% cumulative release, Citrate: ~7% Carboplatin: Water: ~45% cumulative release, PBS and Citrate: 35% cumulative release *Day 7*: Paclitaxel: Water: ~11% cumulative release, PBS ~11.5% cumulative release, Citrate ~10% cumulative release Carboplatin: Water: ~50% cumulative release, PBS: ~30% cumulative release, Citrate: ~25% Day 10: Paclitaxel: Water and PBS: ~15%, cumulative release Citrate: ~12% cumulative release Carboplatin: Water: ~55% cumulative release, PBS: ~35% cumulative release, Citrate: ~30% cumulative release	[39]
Methotrexate	PLA	500 mg porous implant samples Medium: 5 mL of PBS Incubation temperature: 37 °C Shaken in a centrifuge tube at 100 rpm Released amount of drug was determined by UV spectrophotometry	0.5% drug loading: Day 1: ~25% MTX released Day 7: ~50% of MTX released Day 30: ~80% MTX released 1.5% drug loading: Day 1: ~25% MTX released Day 7: ~50% of MTX released Day 30: ~80% MTX released 2.5% drug loading: Day 1: ~25% MTX released Day 7: ~50% of MTX released Day 30: ~80% MTX released All 3 MTX scaffolds demonstrated sustained release A cast 0.5% drug-loaded PLA/MTX scaffold also were examined for 5 days: Similar release pattern but slower release rate	[46]
Ibuprofen	PCL, CS	Samples with two different structures of the same size and different drug contents Medium: 100 mL of PBS (pH 7.4) Incubation temperature: 37 °C Shaking at 100 rpm Sample volume: 100 mL of PBS buffer was changed within specific time Released amount of drug measured by double-beam UV spectrophotometry	Structure 1 (dense): cumulative release in 120 h: 43% Structure 2 (loose, interlaced middle layer): cumulative release in 120 h: 62.3% Structure 2 had higher relative specific surface than Structure 1, which resulted in a more efficient release. Influence of drug and CS content with Structure 2: Drug content (%*w*/*w*) 4%: 28% cumulative release 20%: 100% cumulative release CS content (%*w*/*w*) 4%: 55% cumulative release 20%: 100% cumulative release	[41]
Rifampicin, Dipyridamole	TPU	3D-printed rod samples with different drug concentrations (20 × 3 × 3 mm) The drug release studies of the two drugs were conducted separately. Medium: 1 mL (90%*w*/*w* PBS and 10%*w*/*w* T80) Incubation temperature: 37 °C Sampling: The rods were removed at predetermined 24 hourly intervals and placed in new media.	Rifampicin: Sample 0.1%*w*/*w* and 0.25%*w*/*w*: Low release rate ~8.5% by day 80 (was not discussed further in detail) Samples 0.5%*w*/*w* and 1.0%*w*/*w*: First phase: quicker release during the first 1520 days (close to a linear line) ~55.8% cumulative release Second phase: 2080 days: Samples showed sustained release profile, by day 80~10% cumulative release Conclusion: The samples containing higher drug concentrations are capable for sustained release Dipyridamole: 0.5%*w*/*w*: Showed lower drug release. ca. after 5 days, the sample showed steady release rate up to ~37 days compared to rifampicin 0.5%, dipyridamole showed lower drug release suggesting different interactions with TPU. 1.0%*w*/*w*: After 55 days, sample released ~9% of the initial dipyridamole loading. 1.5%*w*/*w*: Showed higher drug release ca. 8% of initial dipyridamole, however the increase was not 50% higher compared to dipyridamole 1.0% sample.	[40]

## Data Availability

No new data were created or analyzed in this study. Data sharing is not applicable to this article.

## Abbreviations

The following abbreviations are used in this manuscript:

3D	Three-Dimensional
API	Active Pharmaceutical Ingredient
CAD	Computer-Aided Design
CMA	Critical material attribute
CPPs	Critical process attributes
CS	Chitosan
CT	Computed Tomography
DSC	Differential Scanning Calorimetry
EDX	Energy dispersive X-ray
FDM	Fused Deposition Modeling
FTIR	Fourier Transform Infrared Spectroscopy
FRAME	Framework for Regulatory Advanced Manufacturing Evaluation
GABA	Gabapentin
HME	Hot-Melt Extrusion
IDDS	Implantable Drug Delivery System
ISO	International Organization for Standardization
IUD	Intra Uterine Device
MeltPrep	Melt Preparation
MicroCT	Microcomputed tomography
MRI	Magnetic Resonance Imaging
MTX	Methotrexate
MβCD	Methylβ cyclodextrin
NIR	Nuclear magnetic resonance spectroscopy
PBS	Phosphate-buffered saline
PC	Polycarbonate
PCL	Polycaprolactone
PEG	Polyethylene glycol
PETG	Polyethylene terephthalate glycol
PLA	Polylactic acid
PLGA	Poly lacticcoglycolic acid
PMMA	Poly (methyl methacrylate)
PRXD	Powder X-ray diffraction
PVA	Polyvinyl alcohol
QbD	Quality by Design
SEM	Scanning Electron Microscopy
STL	Standard Tessellation Language
Td	Decomposition temperature
Tg	Glass transition temperature
TG/TGA	Thermogravimetric Analysis
Tm	Melting temperature
TPU	Thermoplastic polyurethane
TRELEU	Trehalose and Leucine association
RT-CES	Real time electronic sensing assay
MTT	3-(4,5-dimethylthiazol-2-yl)-2,5-diphenyl-2H-tetrazolium bromide dye
